# Low COVID‐19 vaccination rates in people with severe mental illness and reasons for this: An out‐patient study

**DOI:** 10.1111/acps.13400

**Published:** 2022-03-09

**Authors:** Atheeshaan Arumuham, Oisin O’Brien, Zain Ahmad, Kurosh Nikbin, Oliver D. Howes

**Affiliations:** ^1^ Department of Psychosis Studies Institute of Psychiatry, Psychology & Neuroscience Kings College London London UK; ^2^ South London and Maudsley NHS Foundation Trust London UK; ^3^ Institute of Clinical Sciences (ICS) Faculty of Medicine Imperial College London London UK; ^4^ GKT School of Medical Education King’s College London London UK; ^5^ H Lundbeck A/s St Albans UK

We investigated COVID‐19 vaccination rates in people with severe mental illnesses (SMI) in a sample including two community mental health teams in South London (*n* = 347). Within this cohort, 55% received both doses of the COVID‐19 vaccine, whilst 24% were unvaccinated. In comparison, 75.6% of people in England who were in the same priority group had received both vaccines. On examining the reasons for not having received the vaccine, 21% reported side effect concerns, whilst 20% reported waiting for additional support to organise a vaccine appointment. A further 8.9% reported not having received notification that they were invited to receive the vaccine. These findings suggest that a targeted approach should be taken to increase vaccine uptake in people with SMI.

People with severe mental illnesses (SMI), such as schizophrenia or bipolar disorder, have a worse prognosis following COVID‐19 infection compared with the general population,^1^ highlighting the importance of COVID‐19 vaccination for this group. In response, the UK government included people with SMI in an early priority group to receive COVID‐19 vaccination, as part of the phased roll‐out of COVID‐19 vaccination. This early priority group also included people with other underlying health conditions associated with a poorer COVID‐19 prognosis including cardiovascular disease, respiratory illnesses and diabetes. The programme was led by primary care services, which contacted all patients to offer vaccination against COVID‐19. To ensure convenient and equitable access to vaccines, people could attend community vaccination centres, local vaccination services (local general practitioner (GP) practices and pharmacies) and hospital hubs with or without an appointment. Vaccination with either the Pfizer, Astra‐Zeneca or Moderna vaccines was offered free to all eligible people. However, symptoms (such as paranoia and amotivation) and healthcare inequalities (such as poor integration of mental health and primary care services leading to difficulty in accessing primary care) that affect people with SMI living in the community may act as barriers to accessing COVID‐19 vaccines.^1^, ^2^ In view of this, we hypothesised that COVID‐19 vaccination rates would be lower in people with SMI relative to other groups offered vaccination at the same time. However, vaccination rates in this group are unknown. To address this, we examined vaccine uptake of people with SMI living in the community and explored reasons for not receiving vaccination.

We performed a cross‐sectional study that was approved by the Lambeth Directorate of South London and Maudsley NHS Foundation Trust (SLaM), which included reviewing clinical notes and contacting patients to clarify vaccination status and question reasons for refusal of the vaccine. This audit involved reviewing the electronic primary care and hospital care records of patients against national vaccine guidelines; therefore, patient consent was not required. We reviewed the records of 347 patients from two adult community mental health teams in London, England, to determine demographic characteristics, diagnosis and COVID‐19 vaccination status on 29 June 2021. Where vaccination status was not recorded, we contacted patients to check this (*n* = 31 (9%) were uncontactable) and to ask the reason for not having received the vaccine using a standardised questionnaire: (Q1) ‘Have you been offered a COVID‐19 vaccine?’, (Q2) If Q1 Yes, ‘Have you received a COVID‐19 vaccine?’, (Q3) If Q1 or Q2 No, ‘What is the reason you have not received a vaccine?’. This audit followed the Strengthening the Reporting of Observational Studies in Epidemiology (STROBE) reporting guideline.

COVID‐19 vaccination was offered free to all people with SMI in England from 16 February 2021. Table 1 shows that more than four months later, only 55.0% of people with SMI were confirmed to have received two vaccine doses. In comparison, 75.6% of people in England also offered vaccination from 16 February 2021 being in the same priority group as patients with SMI had received two doses by the same timepoint.^3^ When considering the effect of ethnicity, white patients had higher rates of vaccination than black patients (*X* ^2^ (3239) = 22.889, *p* < 0.001; insufficient data for other comparisons), similar to findings in the general population.^4^ There was no significant difference in vaccine uptake between males and females. Exploratory logistic regression analysis of age, gender, ethnicity and diagnosis found only age associated significantly with vaccination status (data available from authors). Of the 84 people who had not received a vaccine, 56 were contactable. The most common reasons for not having received the vaccine were side effect concerns (21.0%) and waiting for support to arrange vaccination (20.0%). Interestingly, 8.9% of patients reported they had not been invited to receive a vaccine from primary care services, which were tasked with contacting all patients in this group to offer vaccination.

**TABLE 1:** Patient demographic and vaccination status

Patient characteristic	*N* = 347
Age (years), Mean (SD)	46·09 (14·02)
Gender, Male: Female (%)	217:130 (62.5%:37.5%)
Diagnosis, *n*/*N* (%)	
Schizophrenia	220/347 (63·4%)
Schizoaffective disorder	52/347 (14·9%)
Affective disorder	44/347 (12·7%)
Schizophreniform disorder	19/347 (5·5%)
Other disorder	8/347 (2·3%)
Personality disorder	4/347 (1·2%)
Vaccination status, *n*/*N* (%)	
Fully vaccinated	190/347 (55%)
Known unvaccinated	84/347 (24%)
Partly vaccinated	42/347 (12%)
Unknown status	31/347 (8·9%)
Ethnic group, *n*/*N* (%)	
White	121/347 (35%)
Black	118/347 (34%)
Other	61/347 (18%)
Asian	36/347 (10%)
Mixed	11/347 (3·2%)
Reasons for not having received vaccination, *n*/*N* (%)	
Side effect concerns	12/56 (21%)
Interested, awaiting support	11/56 (20%)
Did not elaborate	10/56 (18%)
Other	8/56 (14%)
Does not believe necessary	6/56 (11%)
Not offered vaccine	5/56 (8·9%)
Symptomatic/Suspected COVID	4/56 (7·1%)
Unable to contact from known unvaccinated	28

Given the poorer prognosis from COVID‐19 infection in people with SMI, our findings that a large proportion (up to 45%) of people with SMI is not fully vaccinated against COVID‐19 are concerning.

Some patients (*N* = 28) identified as not having received the vaccine were uncontactable; so, whilst there was no record of them having received a vaccine, it remains possible they had received it. However, even if one assumes all uncontacted patients were fully vaccinated, rates of vaccine uptake in people with SMI (64% if all uncontactable patients were vaccinated) remain lower than in the comparable population offered vaccination at the same time (75.6%).

Although studies exploring vaccine uptake in SMI have borne mixed findings, with high regional variability, a recent study found that a targeted vaccination programme for patients in the community with SMI showed similar rates of vaccine acceptance to the general population, indicating people with SMI accept vaccination when given support to access it.^5^ This suggests that our findings are not primarily because of reluctance to receive a COVID‐19 vaccine amongst people with SMI. Our finding that 20% of the patients who had not received a vaccine were waiting for support and 8.9% of patients were not yet invited to receive the vaccine is consistent with this. These findings suggest that a support programme for SMI patients living in the community, taking into consideration ethnic differences in reasons for vaccine hesitancy, may be beneficial in increasing vaccine uptake in this vulnerable group.

## CONFLICT OF INTEREST

AA, OO, ZA and KN report no grants and have no conflicts of interest.

## Supporting information

Supplementary Material

Supplementary Material

## Data Availability

The data that support the findings of this study are available from the corresponding authors, AA and ODH, upon reasonable request.
