# Supplementary figures and images for: Low COVID‐19 vaccination rates in people with severe mental illness and reasons for this: An out‐patient study
Source: Acta Psychiatr Scand. 2022 Mar 9;145(4):416–8. doi: 10.1111/acps.13400 (PMC9111255; doi:10.1111/acps.13400)

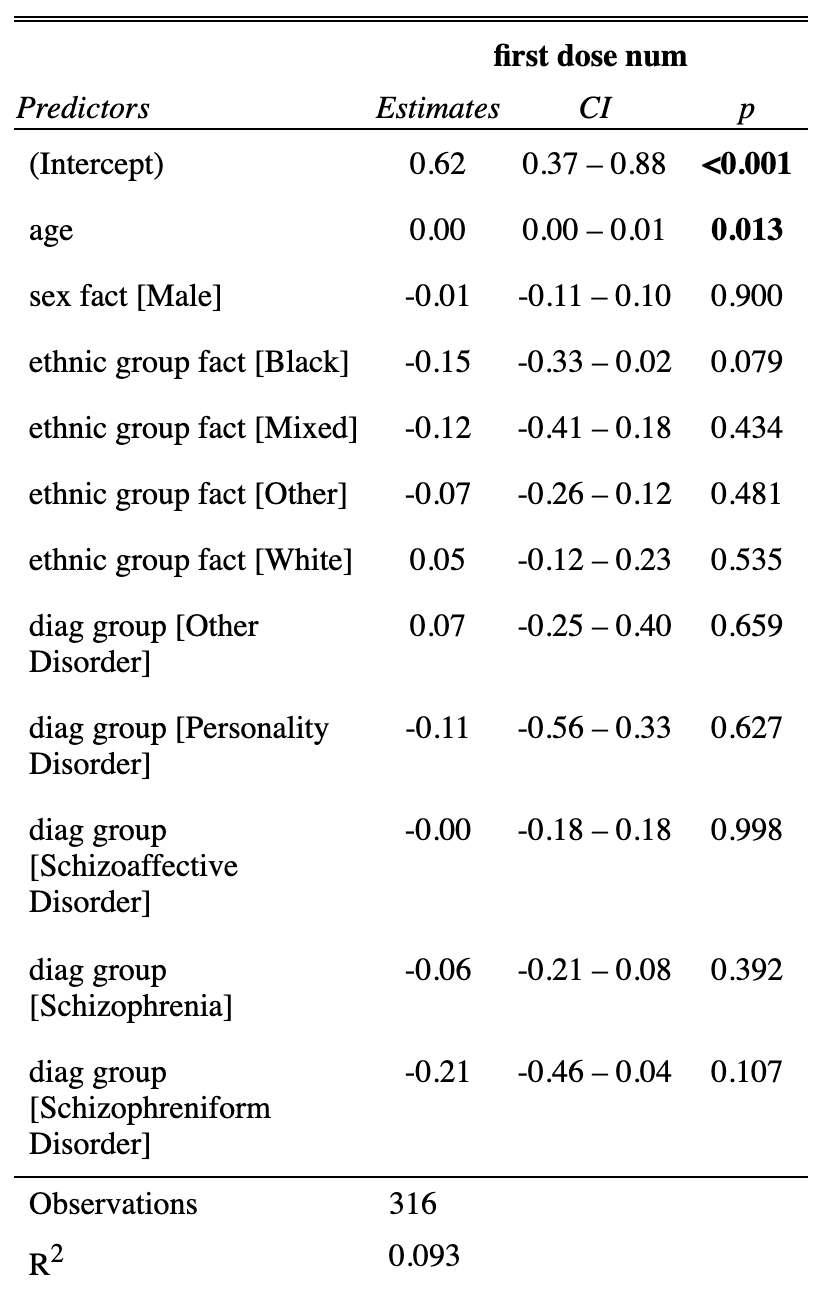

Supplement: Supplementary file 1 — Supplementary Material [file ACPS-145-416-s001.png]

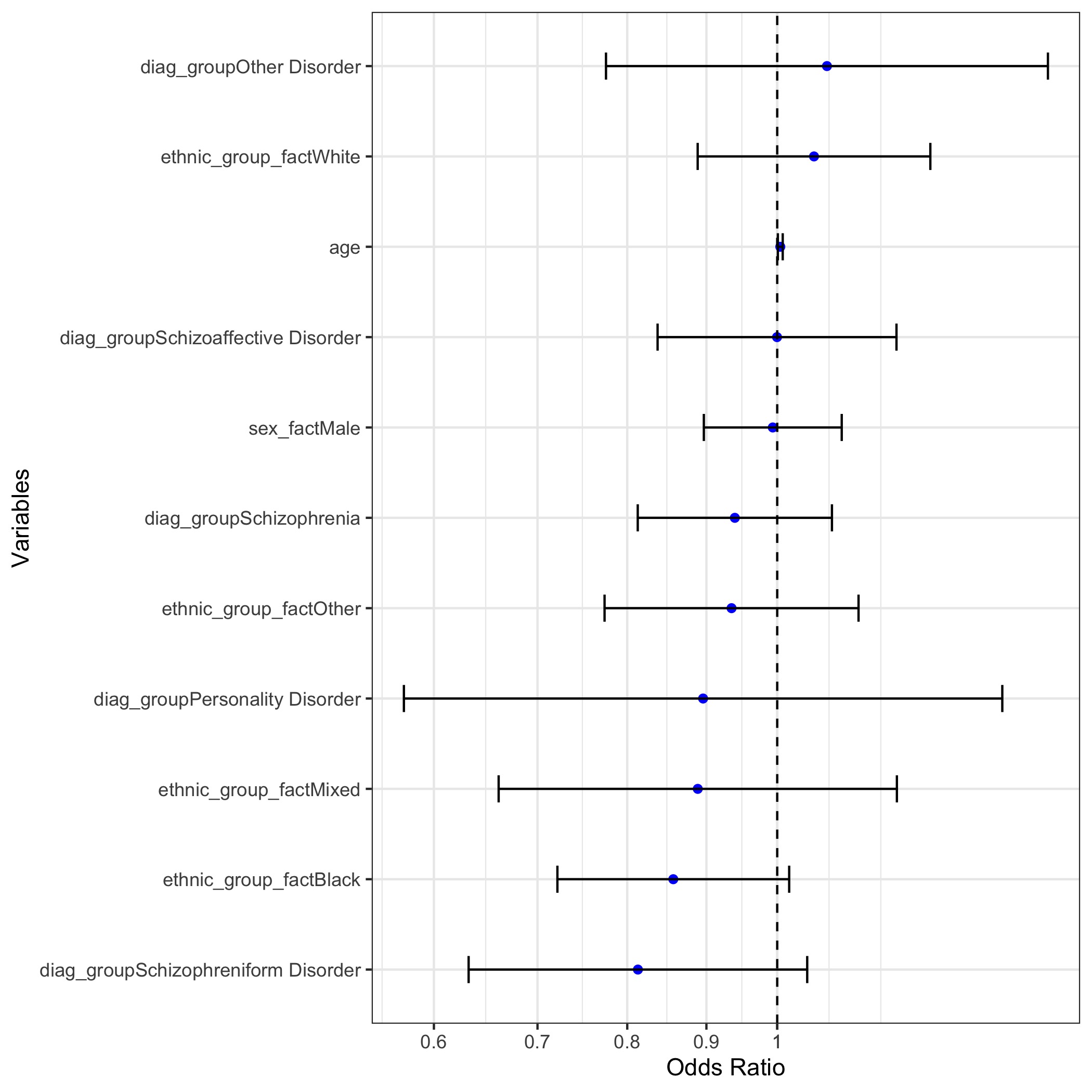

Supplement: Supplementary file 2 — Supplementary Material [file ACPS-145-416-s002.jpg]
